# Body-part compatibility effects are modulated by the tendency for women to experience negative social comparative emotions and the body-type of the model

**DOI:** 10.1371/journal.pone.0179552

**Published:** 2017-06-20

**Authors:** Eva Pila, Kimberely Jovanov, Timothy N. Welsh, Catherine M. Sabiston

**Affiliations:** Faculty of Kinesiology and Physical Education, University of Toronto, Toronto, Canada; Universita degli Studi di Udine, ITALY

## Abstract

Although exposure to physique-salient media images of women’s bodies has been consistently linked with negative psychological consequences, little is known about the cognitive processes that lead to these negative effects. The present study employed a novel adaptation of a computerized response time (RT) task to (i) assess implicit cognitive processing when exposed to the body of another individual, and (ii) examine individual differences in social comparative emotions that may influence the cognitive processing of human bodies. Adult females with low (*n* = 44) or high (*n* = 23) tendencies for comparative emotions completed a task in which they executed responses to coloured targets presented on the hands or feet of images of ultra-thin, average-size, and above average-size female models. Although the colour of the target is the only relevant target feature, it is typically found that the to-be-ignored location of the target on the body of the model influences RTs such that RTs are shorter when the target is on a body-part that is compatible with the responding limb (e.g., hand response when target was on hand) than on a body-part that is incompatible with the responding limb (e.g., hand response when target was on foot). Findings from the present study revealed that the magnitude of the body-part compatibility effect (i.e., the index of the cognitive processing of the model) was modulated by tendencies for affective body-related comparisons. Specifically, women who were prone to experiencing social comparative emotions demonstrated stronger and more consistent body-part compatibility effects across models. Therefore, women with higher social comparison tendencies have heightened processing of bodies at a neurocognitive level and may be at higher risk of the negative outcomes linked with physique-salient media exposure.

## Introduction

Acute exposure to media-portrayed images of body ideals in women has been consistently associated with negative psychosocial outcomes, including negative affect [1], low self-esteem [2], body dissatisfaction [3], disordered eating [4], and depressive symptoms [5]. Cumulative exposure to media representing ultra-thin bodies impacts normative perceptions of body size and perpetuates the normative discontent that women experience around their bodies [6]. The powerful impact of repeated and chronic exposure to media images has prompted significant research attention to examine the outcomes of viewing ultra-thin ideal images [6,7]. Although meaningful advancements have been made in understanding the perceptual, affective, and behavioral outcomes of physique-salient image exposure, very little attention has been paid to understanding the underlying cognitive and associated neural processes. Exploring the neuro-cognitive mechanisms that generate these outcomes is necessary to understand the effects that exposure to physique-salient images has on psychological functioning. This research focus is critical for identifying risk factors of individuals and subsequently developing interventions to mitigate and prevent consequences of exposure.

The importance of examining cognitive and neural mechanisms underlying exposure to media images has been highlighted and several insightful studies have recently been conducted [8,9]. For example, in a neuroimaging study, Uher and colleagues [9] identified a neural network responsible for processing one’s own body and the bodies of others—including the lateral fusiform gyrus, inferior parietal cortex, and lateral prefrontal cortex. Building on this work, Friederich and colleagues [8] describe a similar neural network, and also include a related affective network (i.e., consisting of the anterior cingulate, insula, and amygdala), which is activated if the physique-salient stimuli is self-relevant or emotionally significant to the individual. Overall, this work suggested that social comparison processes may be responsible for activating the neurocognitive networks associated with anxiety and other emotional responses after exposure to physique-salient images. Thus, the manner in which one person processes another person’s (i.e., a model’s) body with respect to the observer’s perception of their own body may be one important factor that shapes the outcomes to exposure. The present study explores this potential cognitive mechanism that might be associated with these neural networks.

In visual cognitive and spatial attention research, some theorists suggest that simply being exposed to an image of a body and attending to a particular body part can increase perceptual sensitivity in the viewer’s own body [10–12]. The observation that merely viewing the body of another can lead to the activation of neurocognitive mechanisms in the self are also supported by tenets of social comparison theory [13]. Specifically, it has been suggested that comparative processes are likely spontaneous, automatic and not entirely under voluntary control [14,15] and the automatic nature of appearance-focused social comparisons has been reported in acute exposure studies [16]. In their study, Henderson-King and colleagues [16] examined the influence of social context on women’s reactions to ideal images in the presence of male confederates who were evaluating the images, and found that viewing ideal bodies did *not* have an effect on self-evaluations. The authors rationalized that women typically “process and react to media imagery on a nonconscious level” (pg. 1410), and that social environmental stimuli (e.g., commentary from males), may curtail the negative automatic effects of exposure to ideal images. Based on these findings, it is possible that appearance-focused social comparisons begin automatically through the activation of neural mechanisms that are involved in processing other peoples’ bodies with reference to the internal representation of the observer’s own body.

Consistent with and to support the notion of an automatic self-other comparison system, researchers from the fields of neuropsychology and neuroscience [17,18] suggest that humans possess an internal, neural representation of the human body, otherwise known as a body schema. Despite considerable debate over the definition of body schema in the neuroscience literature [19] for the purposes of this study, the body schema is conceptualized as an internal representation of one’s own body that enables one to know where their own body is in space, and is also activated when the person observes another human body form [11,20,21]. Thus, it is possible that activation of this representation when one observes another person represents an initial stage of “body-part resonance” or an implicit “self-other matching” process. In this process, the mere observation of another person’s body or body parts activates neurocognitive representations used to understand the person’s own body and those of other people relative to their own body. Although this self-other matching and body resonance mechanism is likely to be largely automatic and stimulus-driven (i.e., the body schema is activated whenever an observer looks at another body), there is evidence that the degree of activation and resonance (from both a cognitive and neural perspective) maybe modulated by a number of factors. These factors include the visual characteristics (self- vs other body, first vs. third person perspective) and species of the observed body [20,22]

Behavioral evidence supporting this embodiment and resonance phenomenon has come from studies of what is known as the *body-part compatibility effect* [12,21,23,24]. In one method of testing the body-part compatibility effect developed by Bach et al. [12], participants were presented with an image of a human body with a colored target stimulus on one limb (e.g., red or blue dot on the hand or foot of body). Participants were instructed to, regardless of where (on which limb) the target stimulus is presented, execute hand responses if the stimulus is red, or foot responses if the stimulus is blue. Thus, the color of the stimulus is the task-relevant feature and the location of the stimulus is a task-irrelevant feature that can and should be ignored. As such, any influence that the task-irrelevant body feature has on response times to the target stimulus is thought to reflect any automatic and involuntary processing of the body-related information. Studies using this task [12] have consistently revealed that response times are shorter on body-part compatible trials where the responding effector and location of the color stimulus on the model is the same (e.g., when the red stimulus is on the hand of the image). This is compared to incompatible body-part trials where the body parts observed and used are different (e.g., when the red stimulus is on the foot of the image). This effect is thought to occur because the observation of the body of the model on the screen will activate the body schema within the observing participant. Further, presenting the stimuli over a specific body part (e.g., the hand) will draw attention to the body part and excite the representation of that specific body part in the observer’s body schema to a greater degree than the representations of the other body parts. This robust activation of the specific body part in the body schema subsequently has a downstream effect that enhances the efficiency with which responses with that limb (e.g., hand) are planned and executed, relative to responses from another non-compatible body part (e.g., the foot). Thus, when the target (e.g., red) for a given response (e.g., hand press) is presented over a model, response times are shorter when that target is presented in a body-part compatible location (e.g., red target on the hand) than when the target is presented on a body-part incompatible position (e.g., red target on the foot). In the body-part incompatible case, the activation associated with the foot would need to be overcome to be able to respond with the hand. Thus, it is thought that the body-part compatibility effect provides evidence that humans match the bodies and body parts of other individuals on to their own neurocognitive body representation when exposed to the body of another individual [12,21].

Drawing from both neuropsychology [18] and social comparison literatures [7,14], it is possible that the magnitude of these automatic body-part resonance mechanisms are modulated by both lower-level features of the body and higher-level cognitive processes. For example, the presence and magnitude of the body-part compatibility effect varies based on the observable characteristics of the images over which the stimuli are presented (e.g., age of model in image [25]; human versus animal models [21]) and by experience and learning [26,27]). Although never previously tested, it is possible that the presence and magnitude of the body-part compatibility effect may be modulated by body-related characteristics in a presented image (e.g., body shape, size, weight). The characteristics of the model might moderate body-part compatibility effects because these characteristics might be “more” or “less” similar to the body characteristics of the observer and, hence, modulate the degree to which the observer’s body schema is activated when observing a given body. Additionally, individual differences in the tendency to engage in social comparison processes may also regulate the presence and magnitude of the self-other matching process. In sum, the activation of the body schema when observing another person’s body might be modulated by the similarity between the body characteristics of the observed and observer’s body, and the tendency with-which the observer engages in physique-related social comparisons. Because the body-part compatibility effect is thought to be a manifestation of the activation of the body schema and subsequent self-other matching process, it is possible that the magnitude of the body-part compatibility effect will be modulated by the individual’s tendency to engage in social comparison and the body-type that the individual is observing.

Social comparison tendencies have been identified as important factors that modulate women’s psychological responses when exposed to physique-salient images [16,28–30]. However, the current conceptualizations of social comparison tendencies are restrictive, in that they do not consider associated affect and emotion—components that have been identified as overlooked but highly relevant factors of social comparison processes [31]. For example, Sabiston and Pila [32] characterized concurrent experiences of body-related envy and shame as emotional domains of social comparisons, which uniquely contribute to women’s body-related experiences. Considering that women who are predisposed to body-related shame (i.e., inadequacy of the self; [33]) and body-related envy (i.e., inferior self, compared to another; [34]) are more threatened by exposure to physique-salient cues [35,36], predisposition to socially comparative emotions may impact efficiency of cognitive processing and the overall resonance and activation of the body schema. Especially considering the emotion-focused neural pathways that are activated by physique-salient body recognition [8,9], this understudied affective dimension of social comparison may contribute to modulating more basic neurocognitive processes such as the body-part compatibility effect.

The main objective of this study was to assess dispositional social comparative emotions as potential modulators in the processing of images women’s bodies via the body-part compatibility effect (i.e., top-down modulation of the body-part matching process). A secondary objective was to examine differences in the magnitude of body-part compatibility based on the type of unique image presented (i.e., bottom-up modulation of the body-part matching process). To this end, we examined differences in the magnitude of body-part compatibility effects in low versus high comparative emotion groups when viewing images of varying body types. It was predicted that dispositions of body-related envy and shame would moderate the physique-salient presentation and body-part compatibility task. Specifically, it was hypothesized that women with higher tendencies for social comparison will be more highly attuned to physique-salient cues and therefore have a more efficient and highly activated resonance process leading to stronger and more consistent body-part compatibility effects. Meanwhile, women with lower social comparison tendencies were expected to demonstrate less efficient and more weakly activated resonance processes leading to weaker and less consistent body part compatibility effects (Hypothesis 1).

Additionally, given that body-part compatibility shows different magnitudes based on the type of physique-salient image presented [21,25], it was hypothesized that the body-part compatibility effect may emerge in varying magnitudes across images of women with different body types (Hypothesis 2). Because this is the first study to examine the body-part resonance process and social comparison using this empirical approach, past methodological recommendations [14] were utilized, whereby several unique model body types across a range of body-types were used (i.e., ultra-thin, average, and above-average models) to explore the potential moderating effect of body type. No specific a priori predictions were formed regarding how each body-type would be processed and how this processing might be modulated by (i.e., interact with) the individuals’ propensity to engage in social comparison.

## Methods

### Participants and procedures

Sixty-seven female participants between the ages of 18 and 25 years (*M*_age_ = 19.97 ± 1.56) from the University of Toronto community volunteered for the study. The research protocol was approved by University of Toronto Research Ethics Board, and all participants provided written informed consent. Participants identified as Caucasian (49.3%), Asian (32.8%), South Asian (7.5%) and Black (6.0%) and mixed-race (4.5%). The study was advertised as a “Study of Attention & Image Perception” and thus participants were naïve to the purpose of the study. Because participants were assigned to low and high social comparison groups based on the results and analysis of questionnaires completed at the end of testing, recruitment continued until there was a high degree of confidence that there would be at least 20 participants in each group. A sample of 20 per group was based on past sample size criteria in body-part compatibility studies [12,21]. Participants were financially compensated with $10 upon completion of the study.

After initial screening for inclusion criteria (i.e., identify as female, age 18 to 25, normal or corrected-to-normal vision, right hand dominant), participants were scheduled to take part in the study. Once at the laboratory and informed consent was received, participants begun the computerized experimental task, then completed a series of web-based surveys. For the experimental task, participants sat in a chair at a desk approximately 70cm away from a 23” LCD computer screen on which all stimuli were presented. Stimuli consisted of physique-salient digital images of three models. One of the three model images was randomly presented on each trial. Models had distinct body shapes consisting of an ultra-thin model, average-size model, and an above average-size model. A series of images were selected from online sources by the primary author based on conceptualizations of media-portrayals of women of different shapes and sizes and informed by past studies employing similar protocols [37–40]. Criteria for images included: i) full front body-shot of model, (ii) two-piece bathing suit, with stomach area, arms and legs fully exposed, and (iii) neutral-pleasant facial expression. Within the bounds of these criteria, a variety of 5–10 photos for each of the three model types was selected by the primary author. Then a discussion between all authors followed to determine the model image that most appropriately represented the media portrayed classification for each body size. The authors discussed until consensus was reached. In a follow-up pilot test, an independent sample of female participants ages 18 to 25 years (*n* = 5) ranked the images on their representation of varied media portrayals of women’s bodies. Unanimously, raters identified each image with the appropriate media-portrayed body size classification (i.e., ultra-thin model, average-sized, above average-sized model). Model type (i.e., ultra-thin, average size, above average size) was a within-subjects independent factor in the main study.

Following confirmation of the physique-salient images, digital manipulation was then used on the images as necessary to match skin, hair and bathing suit color, and to remove the model from the background images and position the model near the centre of the white background. The images were also resized as necessary to ensure the models were approximately the same height (10.4 cm) when presented on the screen during the trials. Then 4 copies of the images were made and a single target stimulus was drawn in each picture. The stimulus was a blue or red circle (2.5cm diameter) that was superimposed over the hand or foot [12,21]. In the experimental task, the blue and red circles were presented equally on the foot and hand locations and were presented simultaneously with the model image on every trial. Further, to prevent an anticipatory attentional shift from the fixation cross, the image was also equally presented in normal and horizontally-flipped orientation such that the red and blue targets were presented equally often on the hand and the foot, and on the left and right side of the body. Hence, in total, there were 8 images for each model type consisting of the factorial combination of colour (blue/red), location (hand, foot), and side (left/right).

Throughout a block of trials, participants placed the right foot over a pedal and the right thumb over a button in a press unit held in the right hand. Participants were told to press the button with the thumb or the pedal with the foot as soon as possible after recognizing that a red or a blue circle, respectively, was presented in the picture. Participants were told to ignore the location of the target and respond only to the colour of the target. Instruction screens were presented at the beginning of each testing phase in a white font on a black background. The Responding Limb (i.e., hand or foot) was included as a main factor in the analysis. The Stimulus Location was included as an additional factor and represented the placement of the stimuli on the limb, denoting compatibility of response—a red target on the hand and a blue target on the foot were considered compatible trials, whereas a red target on the foot and a blue target on the hand were considered body-part incompatible trials.

A custom program written using E-Prime (2.0) software controlled the presentation of the experimental stimuli and recorded the timing and identification of the responses. Each participant completed a familiarization session of six trials of randomized images (equal distribution of model images, red and blue stimuli, and foot and hand positions), before completing the testing phase. Each testing phase consisted of five blocks of 48 trials of the choice response task (240 trials). The 48 trials in each block consisted of three instances (one for each model) of the eight trials derived via the factorial combinations of stimuli (red, blue), limb (hand, foot) and orientation (left, right), repeated twice for each of the three models and presented in a random order.

At the beginning of each trial, the word “READY” was presented in the middle of the white screen with black font for 1000 ms. A black fixation cross directed and maintained attention to the middle of the screen during the foreperiod. Model images were presented randomly 1000 to 3000 ms after the presentation of the fixation cross to discourage anticipation. The picture was positioned with respect to the fixation cross such that the hand, foot, and head were roughly equidistant from the centre of the cross.

Following the experimental test, participants were asked to report demographics (i.e., age, ethnicity and education) and a measure of social comparative emotions, followed by ratings of model image characteristics. Consistent with most experimental studies that employ only a post-test measure [7], the decision to assess dispositional psychological variables post experimental task was made in an effort to avoid priming participants to the physique evaluative portion of the study which may confound automatic assessments of response time.

### Measures

#### Social comparative emotions

Self-report questionnaires were used to assess dispositional body-related envy and shame. Shame was assessed using the 8-item Body Shame Subscale of the Objectified Body Consciousness Scale [41], which assesses dispositional tendencies to feel ashamed of the body’s appearance (i.e., *When I am not the size I think I should be*, *I feel ashamed*) and is rated using a 7-point scale (1 = *strongly agree*; 7 = *strongly disagree*). Higher scores represent higher levels of dispositional body shame. Reliability in the present sample was α = .81. Body envy was assessed using a modified body-specific scale of the 8-item Dispositional Envy Scale [42]. Participants rate their proneness to feeling inferior and resentful when comparing their body to a superior other (i.e., *It is so frustrating to see some people who have great bodies/ physiques with little effort*) on a 5-point scale (1 = *strongly disagree*; 5 = *strongly agree*). Higher scores represent higher levels of dispositional body envy. The adapted version of this scale has been used in past research [43,44] and shows strong preliminary psychometric properties. 1 The Dispositional Envy Scale [42] was modified by contextualizing all items to focus on the body/physique (i.e., original item of “I feel envy every day” was modified to “When I think about my body/physique, I feel envy everyday”). The modified scale was validated in a sample of young adults (*n* = 103; *M*_age_ = 21 years) and maintained its original one-dimensional structure. The Cronbach alpha coefficient was *α* = 0.94 and the scale was highly correlated to a 4-item measure of phenomenological envy (*r* = 0.68, p < 0.0001) and the 5-item Physical Appearance Social Comparisons scale [45] (*r* = 0.48, *p* < 0.0001). The reliability in the present sample was α = .91.

#### Image characteristics

Ratings of each model on the basis of perceived similarity and desirability on a scale of 1 (e.g., “*not at all like me”* and *“not at all desirable”*, respectively) to 5 (e.g., “*very much like me”* and *“very desirable”*, respectively). These scores were then used to rank the models and identify the body-type in the physique-salient image that was most similar and desirable to each participant (e.g., if ultra-thin model was rated 5 for similarity, average rated 2 and above-average rated 1, then ultra-thin model was identified as ‘most similar’). Perceptions of similarity to the models were assessed given this is an important index of social comparison [13,30] and to provide additional information of how participants evaluated their own body shape and size relative to the models.

#### Response time

In the body-part compatibility task, response time (RT) was defined as the time period from the onset of image presentation until a thumb button or foot pedal was pressed. RT data for trials on which the participant executed the wrong response (e.g., a hand, instead of a foot response, for a blue stimuli) were eliminated. RTs shorter than 100 ms (anticipation errors) and longer than 1000 ms (inattention errors) were removed from the data set. Finally, RTs greater than 2 standard deviations above the mean for a condition were considered outliers and were deleted. After data were removed, mean RT was calculated across trials.

#### Stimuli

For the factor Stimuli, compatible and incompatible stimuli were always coded with respect to the colour and location of the stimuli. Thus, a red stimulus (which required a hand press) was coded as a “compatible” stimuli when it was presented on the hand of the model and was coded as “incompatible” when presented on the foot of the model. Likewise, the blue stimuli (which required a foot pedal response) was coded as “compatible” when presented on the foot and coded “incompatible” when presented on the hand.

### Preliminary analysis

Based on conceptual evidence that there are individual differences in tendencies to concurrently experience envy and shame [31,34], a cluster analysis of dispositional body-related envy and shame was conducted to identify individual-level co-occurrence patterns of both emotions. Following past recommendations [46,47], a two-step process was followed to identify the number of clusters that fit the data. First, hierarchical cluster analysis using Ward’s linkage method and squared Euclidean distance was conducted to assess the appropriate number of emotions clusters emergent in the data. Agglomeration coefficients and percent change coefficients suggested the use of 2 separate cluster profiles. Second, a nonhierarchical k-means cluster analysis using simple Euclidean distance was conducted specifying a 2-cluster solution and the initial cluster centers that were generated from the hierarchical analysis. Following the cluster membership analysis, independent samples t-tests were conducted to confirm the two groups were significantly different on the facets of interest (i.e., dispositional body-related envy and shame). Chi-square tests were conducted to confirm cluster group differences in percentage of participants reporting perceived similarity and desirability to each model’s physique.

### Main analysis

Response time was submitted to a 2 (Comparative Emotions cluster membership: low, high) by 3 (Model: ultra-thin, average, above average-size) by 2 (Responding Limb: foot, hand) by 2 (Stimulus Location: compatible, incompatible) mixed model analysis of variance (ANOVA). In the ANOVA, the emergent cluster membership of Emotions was used as a between-subjects factor. Meanwhile, Model, Responding Limb, and Stimuli were repeated measures factors. Post hoc analysis of all significant effects involving 4 or more means was conducted using Tukey’s HSD. Alpha was set at 0.05 for all analyses. Mean RT data for each condition are presented in Table 1.

**Table 1:** Mean (SD) of response time (ms) and response errors collapsed across social comparison group.

Outcome	Limb	Ultra-thin Model		Average Model		Above-average Model	
		Compatible	Incompatible	Compatible	Incompatible	Compatible	Incompatible
RT	Foot	563.31 (177.18)	612.37 (188.07)	575.95 (133.48)	604.65 (138.01)	570.50 (162.62)	615.50 (171.71)
	Hand	426.24 (116.53)	472.48 (142.48)	428.20 (105.73)	475.08 (124.81)	436.22 (124.55)	472.88 (142.70)
Error	Foot	0.43 (0.76)	1.19 (1.26)	0.29 (0.58)	1.48 (1.49)	0.37 (0.62)	0.94 (1.17)
	Hand	0.15 (0.44)	1.01 (1.24)	0.28 (0.57)	1.16 (1.46)	0.21 (0.45)	1.13 (1.23)

## Results

### Preliminary results

There were significant differences in body-related envy, *t*(66) = 9.85, *p* < .001, and shame, *t*(66) = 7.51, *p* < .001, between low and high comparative emotion groups confirming group differences on these factors. The first cluster was labeled as ‘low comparative emotion’ (*n* = 44) and was represented by relatively lower body-related envy (*M* = 1.99, *SD* = 1.99) and shame (*M* = 2.93, *SD* = 0.82). The second cluster was labeled as ‘high comparative emotion’ (*n* = 23) and was represented by relatively higher body-related envy (*M* = 3.68, *SD* = 0.58) and shame (*M* = 4.54, *SD* = 0.86). Women in the low comparative emotions group rated the ultra-thin model as most similar (56.8%), followed by the average (38.6%) and above-average model (9.1%) compared to women in high comparative group who rated the average model as most similar (65.2%), followed by the ultra-thin (17.4%) and above-average models (17.4%), χ^2^ (2, 67) = 10.44, *p* < .05. There were no significant group differences between low and high comparative emotions for model desirability (χ^2^ [2, 67] = 0.54, *p* = .76). Overall, women rated the ultra-thin model as most desirable (89.6%), followed by the average model (9.0%), and the above-average model (1.5%).

### Main results

In total, 3% of the trials were deleted as anticipation errors, inattention errors, outliers or wrong responses (e.g., a hand response, instead of a foot response, for a blue stimulus). Levene’s test indicated no violations to homogeneity of variance for any of the hand (*F* = 0.01 to 3.88, *p* = 0.053 to 0.910) or foot (*F* = 0.01 to 2.27, *p* = 0.14 to 0.925) responses, across models. These findings provide support for the acceptability of unequal sample sizes between groups as revealed through the cluster analysis. The analysis of RT revealed main effects of Responding Limb, *F* (1, 65) = 712.00, *p* < .05, η_p_^2^ = 0.92, and of Stimulus Location, *F* (1, 65) = 238.10, *p* < .05, η_p_^2^ = 0.79. RTs for foot responses (*M* = 613 ms; *SD* = 80.1) were longer than those for hand responses (*M* = 474 ms; *SD* = 73.5) and RTs for compatible trials (*M* = 524 ms; *SD* = 72.7) were shorter than those for incompatible trials (*M* = 563 ms; *SD* = 77.4). These results replicate previous findings in body-part compatibility [12,21] and justify further factorial analysis. No other significant main effects (i.e., model), 2-way interaction effects (i.e., model by emotion; stimulus by emotion; respond limb by emotion; model by responding limb; model by stimulus), nor 3-way interaction effects (i.e., model by stimulus by emotion; model by responding limb by emotion; stimulus by responding limb by emotion; model by stimulus by responding limb) emerged.

**Fig 1:**
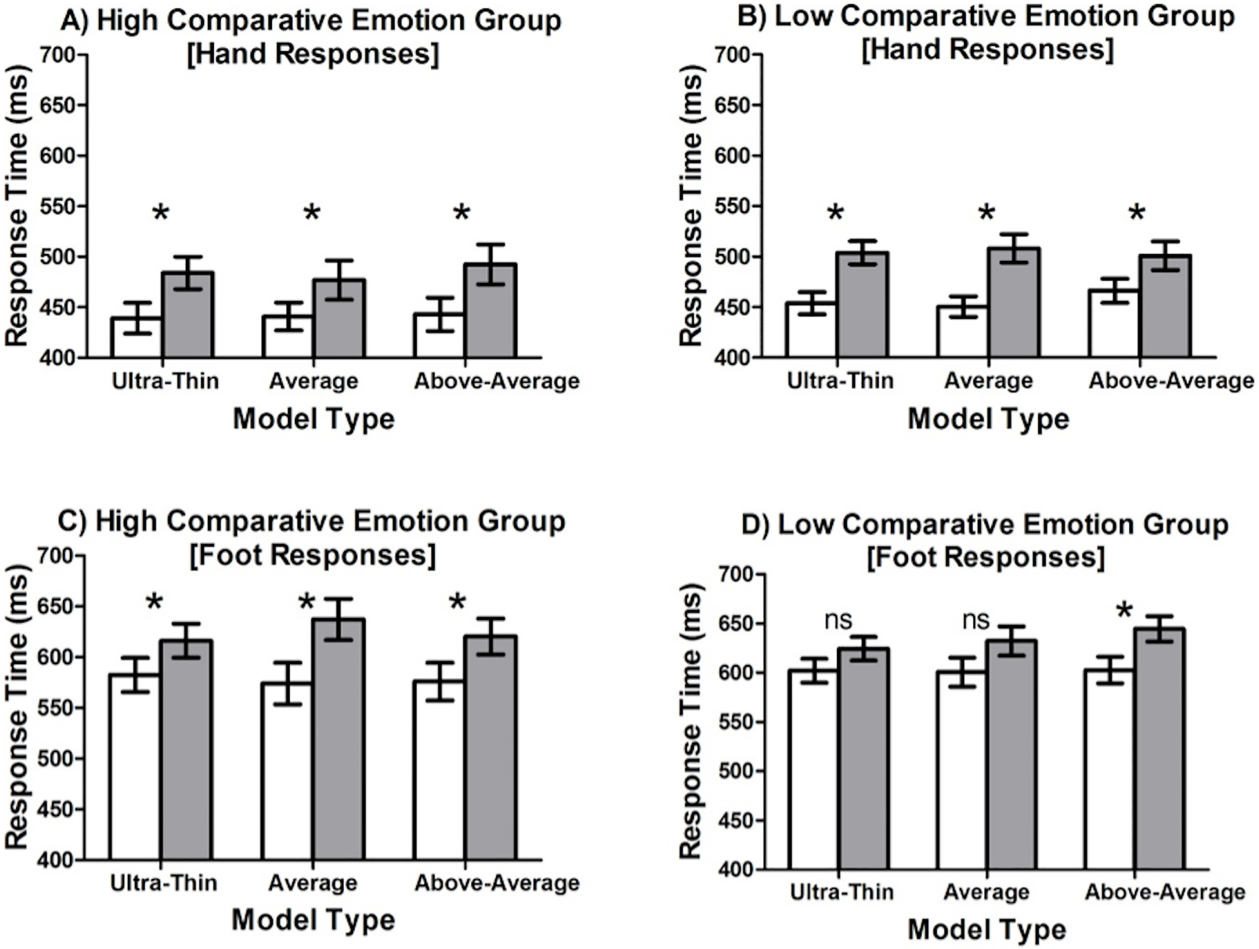
Mean RT (ms) for hand and foot responses in high comparative (A & C) and low comparative (B & D) emotion groups. White bars represent compatible responses and gray bars represent incompatible responses. Standard errors of the compatible and incompatible means are displayed.

The key finding was that there was a significant 4-way interaction between Comparative emotion, Model, Responding Limb, and Stimulus location, *F* (2,130) = 3.22, *p* < .05, η_p_^2^ = 0.047. Post hoc analysis of the RTs revealed that the high comparative emotion group demonstrated significant body-part compatibility effects for all models when either hand or foot responses were required (Cohen’s ds ranging from 0.39 to 0.78; Fig 1a and 1c). For the low comparative emotion group, body-part compatibility effects were significant across all models when hand responses were required (Cohen’s *d*s ranging from 0.39 to 0.70; Fig 1b). For foot responses, the low comparative emotions group had significant body-part compatibility effects only when participants observed the above-average model (Cohen’s *d* = 0.42). No significant body-part compatibility effects were observed in foot responses when the low comparative emotion group responded to stimuli on the ultra-thin (Cohen’s *d* = 0.31) or average model (Cohen’s *d* = 0.27; Fig 1d). Overall, compatibility effects were stronger (i.e., higher mean) and more consistent (i.e., similar means) for the high comparative emotion group than for the low comparative emotion group.

## Discussion

In this novel study, we used an experimental task designed to assess the cognitive processing of the body to better understand how women process physique-salient images. Specifically, the aim of this study was to assess how an individual’s tendency for affective body-related social comparisons affects their processing of images of women’s bodies. Alterations in the self-other matching process were predicted to be indexed by modulations in the strength and consistency of the body-part compatibility effect. Consistent with previous findings [12,21,24,27], the results of the study replicated an overall body-part compatibility effect with images of various model types. That is, RTs were shorter when the location of the target on the limb of the model was the same as the responding limb of the participant (compatible trial) than when the target was presented on a limb that was different from the responding limb (incompatible trial). It is thought that this body-part compatibility effect is a manifestation of the self-other matching process, whereby humans can automatically access body-related schemas when presented with an acute self-relevant image of a body [10,12,21]. The critical new finding of the present study was that this body-part compatibility effect was modulated by the persons’ tendency to experience body-related comparative emotions of envy and shame, and the body-type of the model that was being observed—participants in the high social comparative group showed compatibility with all body-types whereas participants in the low social comparative group only showed consistent compatibility effects with the above-average model. Thus, these findings suggest that the self-other matching process is influenced by both their tendency to experience negative social comparative emotions, and by the body-type being observed.

The first point that we will address is that women’s tendencies to experience body-related comparative emotions were important modulators for the body-part compatibility effect. Specifically, compatibility effects were stronger and more consistent for women with higher tendencies for experiencing social comparative emotions than women with lower tendencies for experiencing these emotions. Based on this finding, it is highly probable that women prone to body-related envy and shame have stronger neurocognitive activations in response to observing the body of another person, as well as more efficient processing of physique-salient cues. That is, the body schema may be more highly and/or consistently activated during exposure to a physique-salient image among women who are more, compared to less, prone to negative social comparative emotions. It is premature to suggest a cause-and-effect relationship or directionality (i.e., whether or not a person is more likely to experience negative emotions *because* they are more likely to have a strong activation of the body schema in self-other matching [a bottom-up stimulus driven effect], or there is stronger activation of the body schema *because* a person is more likely to engage in self-other matching [a top-down priming effect], or both). The present data do, however, indicate that that the self-other matching process is stronger and more consistent in the group of women who are more likely to experience social comparative emotions than those who are not as likely.

These findings are supported by past experimental evidence, whereby acute bouts of envy are linked with more heightened processing of information about targets of comparisons [48]. Therefore, there may be something inherent in the proneness to experience comparative emotions that modulates basic neuro-cognitive mechanisms. Additionally, this finding is supported by recent evidence highlighting individuals with psychological disorders that involve a focus on the body (i.e., anorexia nervosa; body dysmorphic disorder) have higher sensitivity when processing body-related stimuli [49,50]–in particular when exposed to the body of another individual [51]. The current study highlights how the self-other matching or body-part resonance may be another cognitive process that is differentially experienced across individuals, based on higher-level emotional dispositions. Observations from the current study may have important implications for acute exposure to physique-salient portrayals, in that a subset of women have heightened processing at a neurocognitive level, and may be at higher risk for experiencing the well-documented negative consequence associated with media exposure [6,7].

For methodological [14] and theoretical [6] reasons, this experimental design included varied body types to examine if unique body types would be differentially interpreted in terms of body-part compatibility. Partially in support of Hypothesis 2, the strength and consistency of the compatibility effects varied according to model body type and observer. This objective was secondary to the main findings and precludes a specific rationale beyond speculation. Based on the observed difference in relative magnitudes of the body-part compatibility effects, foot responses for high comparative group reflect more efficient processing of the ultra-thin model compared to the average and above-average models. This effect may be illustrating an exposure normative effect, whereby women are consistently exposed to abundant ultra-thin images in the media, leading to more efficient processing [52–54]. Meanwhile, hand responses for the high comparative group may reflect more efficient processing of the above-average model and the average model (to a lesser degree)–which may be associated with the high degree of perceived similarity between the self and the bodies of the average and above-average models, thus reflecting more efficient access to body schemas [25]. In the process of making judgments on attractiveness, eye movements tend to fixate around the mid-section of the observed body, with females emphasizing fixations around the stomach, particularly when the body is larger in size [55]. Among images used in the present experiment, the midsection was arguably the most salient difference in body shapes between the models (i.e., due to visible adipose in the average and above-average models being most predominant feature differentiating these models from the ultra-thin model). Therefore, it is possible that since the hands of the model were close to the stomach region and attention was already drawn and held by the stomach by automatic judgements [55,56], that this made processing of the stimuli on the hand more efficient for the average and above-average models. However, this potential explanation is largely speculative and more research is needed to determine why different parts of the body might be differentially processed and sensitive to body part mapping. Further, before robust conclusions can be drawn, it is unclear if the participant’s actual or objective body weight, size, and shape—beyond perceptual similarity—is influencing these processes. Future research should be conducted to examine both objective measures of body shape and size (i.e., body mass index, waist-to-height ratio), and the degree of perceptual body distortion (i.e., the magnitude of difference between perceived and objective measures of body size/shape, self-reported perceived similarity to model body size/shape), as both may influence cognitive processing via the body-part compatibility effect.

Additionally, hand responses (although contrary to foot responses) for the low comparative women show more efficient processing when exposed to the ultra-thin ideal, which may also reflect a function of perceived similarity (e.g., majority of low comparative women rated the ultra-thin model as most similar). This observed difference in the magnitude of hand and foot responses may be due to the discrete processing functions that have been previously reported for each limb [21,26] and the placement of the hand stimulus near the model’s midsection—which may draw attention to a prominent area of focus and body dissatisfaction in females [57–59], and in light of speculative rationale previously presented in the present study. Due to the speculative nature of these findings, it is imperative for future research to examine the impact of perceived similarity of media-portrayed female body types to elucidate its role in modulating body-mapping processes.

Despite the present study being the first to examine processes related to basic neurocognitive mechanisms and social comparison in an experimental design, findings from the present study should be interpreted with some caution given some limitations. First, the presumption that social comparisons are automatic and may be reflected in the body-part compatibility effect was not directly tested. Future research is needed to examine the specific links between automatic social comparisons and low-level neurocognitive mechanisms that may reflect these processes—studies of the responsiveness in the extrastriate body area (EBA) during the presentation of these different images will be highly informative in this regard. The EBA is an early visual processing area that is highly tuned to the processing of bodies. In particular, it would be instructive to assess any low/high social comparison group differences in the activation of EBA and other areas associated with body processing and self-other matching (e.g., temporoparietal junction (TPJ)) during the perception of different body types. In fact, there is some literature to suggest that women with body-related disturbances and eating disorder symptoms have weaker activation of the EBA [9], and possess altered structural and functional capacities of the EBA [60,61]. As such, social comparative emotional tendencies may be relevant modulators within the EBA—as a critical neural network associated with body image disturbances [62,63]. Second, although all efforts were made to ensure that the main experimentally-relevant feature that differentiated the models was level of “thinness” (i.e., images were matched on the most salient criteria [i.e., pose, clothing, body parts exposed, facial expression, hair color, skin color, etc.]–it is not possible to account for which features of the model participants were comparing in their assessment of similarity between the model’s body and their own body. Future research is needed to examine the extent to which specific components of appearance, beyond body size, may modulate differences in the magnitude of the body-part compatibility effect. Third, the presentation of the model images was bound by efforts to replicate the experimental test methodology (e.g., model presented on white background in upright position), thus raising concern for external validity with regards to how the bodies of models are processed in advertisements and other media. Future experimental studies can extend the current methodology to model images extracted directly from various media sources. Fourth, participants were not screened for conditions that impact perceptual body size distortion (i.e., severe body image disturbances, eating disorder pathology), which may have impacted the responses of the body stimuli. Future research should screen these participants for exclusion. And lastly, the sample of volunteer college-aged females potentially limits generalizability. However, despite potential limitations regarding models and sample characteristics, it is reported that exposure to physique-salient images has damaging consequences regardless of individual differences [64].

Notwithstanding the limitations, this study is a novel exploration of acute physique-salient media exposure via basic neurocognitive mechanisms that may underlie social comparative processes. It is well known that women are exposed to a multitude of physique-related media and social sources that endorse restrictive ideals for body shape, size and weight [7] and perpetuate the normative discontent that women experience [64]. In conjunction with evidence that mere exposure to media portrayed images leads to automatic social comparison processes [15], it becomes imperative to understand the effects on associated neurocognitive processing. Evidence from this initial study indicates that the way women process the bodies of models is influenced by their tendency to experience social comparative emotions and the body-type of the model they are observing. Although it is too early to speculate about the direction of the influence, the data indicate that women who experience negative responses to physique-salient images may do so because the body schema is more highly activated during self-other matching, or because of a priming of their body schema. This new knowledge contributes to our understanding about the processing of exposure to physique-salient images and the array of associated negative consequences—including alterations to neurocognitive processing. These findings may also have important clinical implications in identifying at-risk individuals (i.e., high social comparison tendencies), and then consequently targeting these individuals with intervention strategies to reduce capacities for social comparisons, and mitigate the consequences of exposure to physique-salient images.
